# PTEN Depletion Decreases Disease Severity and Modestly Prolongs Survival in a Mouse Model of Spinal Muscular Atrophy

**DOI:** 10.1038/mt.2014.209

**Published:** 2015-01-06

**Authors:** Daniel Little, Chiara F Valori, Chantal A Mutsaers, Ellen J Bennett, Matthew Wyles, Basil Sharrack, Pamela J Shaw, Thomas H Gillingwater, Mimoun Azzouz, Ke Ning

**Affiliations:** 1 Department of Neuroscience, Sheffield Institute for Translational Neuroscience (SITraN), University of Sheffield, Sheffield, UK; 2 Centre for Integrative Physiology & Euan MacDonald Centre for Motor Neurone Disease Research, University of Edinburgh, Edinburgh, UK; 3 Academic Department of Neurology, Royal Hallamshire Hospital, Sheffield Teaching Hospital Foundation Trust, Sheffield, UK; 4 Faculty of Medicine, King Abdulaziz University, Jeddah, Saudi Arabia; 5 Present address: German Centre for Neurodegenerative Diseases (DZNE), Paul-Ehrlich Strasse, Tübingen, Germany

## Abstract

Spinal muscular atrophy (SMA) is the second most common genetic cause of death in childhood. However, no effective treatment is available to halt disease progression. SMA is caused by mutations in the *survival motor neuron 1* (SMN1) gene. We previously reported that PTEN depletion leads to an increase in survival of SMN-deficient motor neurons. Here, we aimed to establish the impact of PTEN modulation in an SMA mouse model *in vivo*. Initial experiments using intramuscular delivery of adeno-associated vector serotype 6 (AAV6) expressing shRNA against PTEN in an established mouse model of severe SMA (SMNΔ7) demonstrated the ability to ameliorate the severity of neuromuscular junction pathology. Subsequently, we developed self-complementary AAV9 expressing siPTEN (scAAV9-siPTEN) to allow evaluation of the effect of systemic suppression of PTEN on the disease course of SMA *in vivo*. Treatment with a single injection of scAAV9-siPTEN at postnatal day 1 resulted in a modest threefold extension of the lifespan of SMNΔ7 mice, increasing mean survival to 30 days, compared to 10 days in untreated mice. Our data revealed that systemic PTEN depletion is an important disease modifier in SMNΔ7 mice, and therapies aimed at lowering PTEN expression may therefore offer a potential therapeutic strategy for SMA.

## Introduction

The characteristic neuromuscular defects observed in spinal muscular atrophy (SMA) result primarily from the death of motor neurons in the anterior horn of the spinal cord. SMA is caused by mutations or deletion of the telomeric copy of the *survival motor neuron 1* gene (*SMN1*) which results in reduced SMN protein levels.^1,2^ SMN is ubiquitously expressed and is involved in many aspects of RNA metabolism.^3^ It therefore remains unclear exactly why SMN deficiency predominantly affects motor neurons.^4^ One distinctive feature of SMN-deficient motor neurons is an axon elongation defect, which has been reported in cultured cells,^5^ as well as in zebrafish embryos with reduced SMN levels.^6^ In the growth cones of Smn-deficient murine motor neurons, reduced β-actin protein and mRNA levels were also observed.^7^ This suggests that SMN may be necessary for mRNA transport along axons. Moreover, neuromuscular junction (NMJ) defects have been widely reported in a range of animal models of SMA.^7,8,9,10^

We and others have reported that restoring SMN expression^11^ rescues early lethality in a well-characterized mouse model of disease, and this is clearly a promising therapeutic approach for SMA. However, modulating the expression of other genes might provide additional motor neuron protection through alternative mechanisms and molecular pathways. For example, cardiac defects have been reported in a range of animal models of SMA, the severity of which may not be directly related to SMN levels.^12,13,14^ We have therefore focused our attention on the potential for PTEN knockdown to ameliorate disease pathology in SMA, since we first reported that experimental targeting of PTEN increases neuronal survival following ischemic injury *in vitro* and *in vivo*.^15^ Our recent studies have revealed that PTEN depletion in cultured Smn-deficient murine motor neurons ameliorates axon outgrowth defects, increases growth cone size, and improves cell survival.^16^ PTEN negatively regulates the proliferation and size of neural stem cells as well as promoting apoptosis.^17^ This protein exerts its role through the negative regulation of the PI3K/PKB/Akt signaling pathway.^18^ It is expressed in the mouse brain during late development and is preferentially expressed in neurons in the adult brain.^19^ The PTEN protein is also enriched in cell bodies and axon terminals of purified motor neurons.^16^ Depletion of PTEN promotes regeneration of axons in adult retinal ganglion cells and neurites of cortical neurons.^20,21^

Our previous study using focal intramuscular injection of AAV6-siPTEN to silence PTEN in mice *in vivo* has demonstrated successful knockdown of PTEN levels in SMA animals, as well as successful targeting of motor neurons in the ventral horn of the spinal cord.^16^ Here, we initially extended our findings to show that intramuscular injection of AAV6-siPTEN can ameliorate NMJ pathology in SMA mice. We then sought to establish whether silencing PTEN across a broader range of cellular targets could ameliorate the overall SMA phenotype in mice. We therefore generated a scAAV9-siPTEN vector and administered it by intravenous injection to neonatal SMNΔ7 mice to achieve widespread knockdown of PTEN expression. This treatment improved the motor function of SMA mice and significantly extended their lifespan. Histological analysis showed that intravenously administered AAV9 can mediate significant transduction efficiency^11,22^ in the spinal cord, correlating with a significant reduction in PTEN protein level.

## Results

### AAV6-mediated PTEN silencing improves NMJ innervation

**Figure 1:**
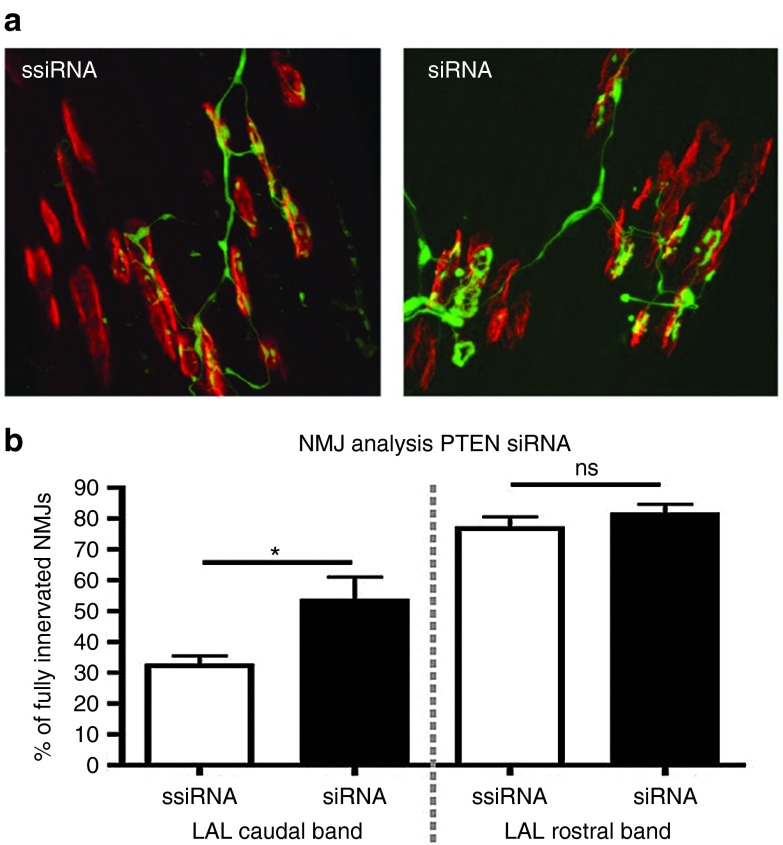
**scAAV6-mediated knockdown of PTEN ameliorates neuromuscular junction (NMJ) pathology in SMA mice.** (**a**) Representative confocal micrographs showing widespread NMJ pathology in the caudal band of the levator auris longus muscle (LAL) in SMNΔ7 mice at postnatal day 10 that received intramuscular injection of scrambled siPTEN (AAV6-ssiPTEN) at postnatal day 1 (left panel) and reduced pathology in mice that received intramuscular injection of siPTEN (AAV6-siPTEN). Antibodies against neurofilament proteins were used to label motor axon collaterals (green), and tetramethylrhodamine-conjugated α-bungarotoxin was used to label acetylcholine receptors at the motor endplate (red). More motor endplates appeared to be innervated by overlying motor nerve terminals in the AAV6-siPTEN–treated muscle. (**b**) Bar chart showing the extent of NMJ denervation (mean ± SEM) in the affected caudal band, and unaffected rostral band, of the LAL muscle in treated (siRNA) and untreated (ssiRNA) mice. This analysis revealed significantly more innervated NMJs in the caudal band of treated mice (*P < 0.05, *n* = 4 mice per group, Student's *t*-test).

Given our previous encouraging data showing that PTEN depletion leads to an increase in survival of SMN-deficient motor neurons *in vitro* and that injection of adeno-associated virus serotype 6 (AAV6) expressing siPTEN into hind limb muscles of mice is capable of targeting PTEN expression in spinal cord motor neurons,^16^ we first wanted to examine whether local targeting of PTEN with AAV6 could rescue effects on muscle denervation in SMA. Previous studies have identified loss of nerve/muscle connectivity at the NMJ as one of the earliest and most important pathological events in SMA.^23,24^ We therefore examined the effect of targeting PTEN on NMJ pathology in *SMN2*^*+/+*^; *SMNΔ7*^*+/+*^; *Smn*^*-/-*^ (SMNΔ7) mice, a well-characterized model of SMA.^25^ We injected 10^10^ vector genome of AAV6-siPTEN (*n* = 4 mice) or AAV6-scrambled-siPTEN (*n* = 4 mice) vectors into the levator auris longus (LAL) muscle of postnatal day 1 SMNΔ7 mice. The LAL muscle was chosen for these experiments as it has a caudal band where NMJ pathology is very pronounced in SMA mice, alongside a rostral band where NMJ pathology is almost entirely absent.^23,26^ Qualitative assessment of NMJs in the caudal band of the muscle suggested a decrease in the numbers of denervated NMJs in the AAV6-siPTEN tissue compared to AAV6-scrambled-siPTEN tissue (**Figure 1a**). Quantitative analysis of NMJ integrity revealed a significant increase in the numbers of innervated motor endplates in the AAV6-siPTEN–treated muscles (**Figure 1b**). Importantly, AAV6-siPTEN treatment had no effect on the remaining healthy NMJs located in the rostral band of the muscle (**Figure 1b**), suggesting that reducing PTEN expression was well tolerated by healthy motor neurons.

### scAAV9-siPTEN improves survival and phenotype of the SMNΔ7 mouse model

**Figure 2:**
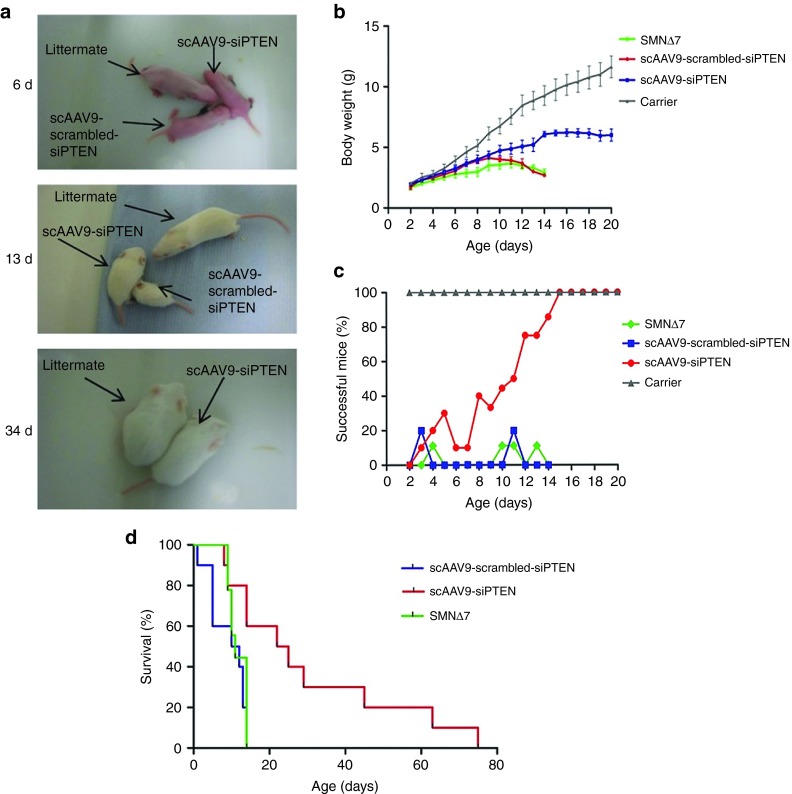
**A single scAAV9-siPTEN injection improves weight gain and extends the lifespan of the SMNΔ**7** mouse model of SMA.** (**a**) Images showing the same SMNΔ7 pups at different ages as indicated; for reference, a gender-matched carrier littermate was included. The pups were injected at postnatal day 1 with either scAAV9-siPTEN or scAAV9-scrambled-siPTEN. (**b**) Body weight increase in SMNΔ7 mice injected with scAAV9-siPTEN or scAAV9-scrambled-siPTEN from postnatal days 2–20. Body weight of scAAV-siPTEN injected mice was significantly greater than that of scAAV9-scrambled-siPTEN mice from day 11 onward (*P* < 0.05 two-way ANOVA with Bonferroni *post hoc* test). Untreated and carrier groups were included as controls. (**c**) Percentage of mice able to complete the righting reflex test. Mice were placed on their backs and were deemed successful if they were able to reorientate within 30 seconds. scAAV9-siPTEN–injected mice improved gradually over the first 15 days, by which time all mice were able to complete the test. (**d**) Kaplan–Meier cumulative survival curves in the different experimental groups. scAAV9-siPTEN–treated mice lived significantly longer than scAAV9-scrambled-siPTEN or SMNΔ7 controls (*P* < 0.01).

To explore the possibility that systemic PTEN reduction might improve survival in SMA, we developed self-complementary adeno-associated virus serotype 9 expressing RNA interference against PTEN (scAAV9-siPTEN; see Materials and Methods). We injected 10 µl (10^10^ vector genome) of scAAV9-siPTEN (*n* = 10) or scAAV9-scrambled-siPTEN (*n* = 10) vectors systemically into the facial vein of postnatal day 1 SMNΔ7 mice. As additional controls, we included untreated SMNΔ7 (*n* = 9) and *SMN2*^*+/+*^; *SMNΔ7*^*+/+*^; *Smn*^*+/-*^ (carrier) mice (*n* = 6) in the study. scAAV9-siPTEN–injected mice showed gradual gain of body weight which continued to increase, whereas the weight of scAAV9-scrambled-siPTEN and untreated SMNΔ7 controls began to fall from postnatal day 9 (**Figure 2b**). The body weight of scAAV9-siPTEN mice became significantly greater than that of scAAV9-scrambled-siPTEN group from day 11 onward. The body weight of scAAV9-siPTEN–injected mice continued to slowly increase throughout their life but remained approximately half the weight of carrier littermates (**Figure 2b**).

Injection of scAAV9-siPTEN resulted in a significant increase in lifespan, with 60% of injected mice surviving longer than any scAAV9-scrambled-siPTEN–injected mice (**Figure 2d**, **Supplementary Videos S1 and S2**). Mean survival for scAAV9-siPTEN–injected mice was 30.4 ± 7.4 days with the longest surviving mouse living for 74 days. In comparison, there was a mean survival of 9.2 ± 1.5 and 10.56 ± 1.42 days for scAAV9-scrambled-siPTEN and untreated SMNΔ7 mice, respectively (**Figure 2d**, **Supplementary Videos S1 and S2**). There was no significant difference in survival between scrambled-siPTEN and SMNΔ7 controls.

### Treated SMNΔ7 mice display improved motor function

The motor function of these mice was assessed daily by performing a righting reflex test. The mice were placed on their backs and given 30 seconds to readjust themselves onto their paws. If they were able to achieve the upright posture within the allocated time, they were deemed successful. The number of successful mice increased steadily in the scAAV9-siPTEN group reaching 100% by 15 days (**Figure 2c**). In contrast, scAAV9-scrambled-siPTEN controls were rarely able to complete the test, with the highest level of success reaching 20% on two nonconsecutive days which was very similar to untreated SMNΔ7 mice. For comparison, carrier littermates were also tested for their ability to self-right; all carriers tested were able to successfully complete the test from day 2 onward. This shows a robust improvement in phenotype of scAAV9-siPTEN–injected mice over time (**Figure 2c**).

The rescued mice were assessed daily for any signs of ill health as well as for body weight and motor function. The tails of the rescued mice were clearly shorter and thicker than usual and began to display necrosis as the animals aged. Furthermore, the ears of these animals became red and inflamed before displaying signs of necrosis too. This phenotype has previously been reported in rescued SMA mice and is thought to be a part of the disease process rather than an effect of the treatment.^27,28,29,30,31^ Furthermore, extremity necrosis has also been reported in two patients suffering from severe SMA.^32^ As the mice aged, they began to display reduced provoked behavior alongside lack of grooming and a very slight reduction in weight and were therefore humanely culled at this point. All mice were still able to perform the righting reflex at this point but did show evidence of hind limb weakness and muscle wasting. It is thus unlikely that the described moribund appearance of the mice was due to motor impairment but rather to some other unidentified cause.

### scAAV9-mediated siRNA delivery reduces PTEN expression in spinal cord motor neurons

**Figure 3:**
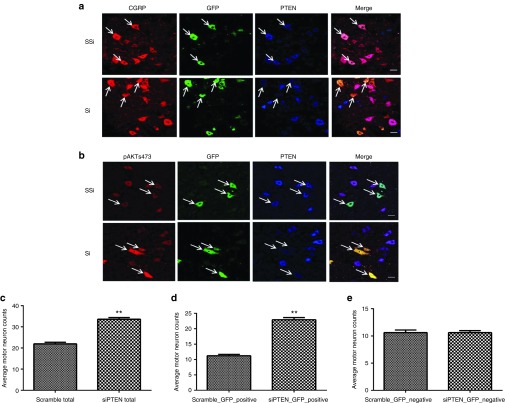
**RNAi-mediated reduction in PTEN expression in SMNΔ7 transgenic mice after scAAV9-siPTEN gene transfer to motor neurons.** Immunofluorescence showing a reduction in PTEN expression in scAAV9-siPTEN-transduced motor neurons as revealed by antibodies against PTEN (blue) (**a,b**), CGRP (red, **a**), and GFP (green, **a,b**) compared with scAAV9-ssiPTEN controls. (**b**) PTEN knockdown results in increased immunolabelling phosphorylated-AKT (red) in scAAV9-siPTEN-transduced motor neurons (green). Bar = 20 µm. scAAV-mediated PTEN knockdown improves motor neuron survival in SMNΔ7 mice. (**c**) Quantification of CGRP-positive cells in siPTEN or scrambled-siPTEN transduced lumbar spinal cord sections, showing the average number of motor neurons per section in 12 sections per animal. (**d**) GFP-positive CGRP neuron count. (**e**) GFP-negative CGRP neuron count. **P < 0.01; *n* = 3; Student's *t*-test.

Systemic injection of high titer AAV9 viral vectors has previously been reported to produce widespread transduction within the central nervous system as well as other internal organs.^22,33^ Immunofluorescence was used to investigate the level of transduction of motor neurons in the lumbar spinal cord and to determine the efficiency of the vector for PTEN silencing. The siRNA sequence used has been previously reported to successfully silence PTEN both *in vitro* and *in vivo*,^16^ the construct also expresses green fluorescent protein (GFP) under a cytomegalovirus promoter to enable identification of transduced cells. Immunofluorescence revealed transduction of motor neurons within the lumbar spinal cord as demonstrated by GFP-positive cells in both scAAV9-siPTEN and scAAV9-scrambled-siPTEN–injected animals (**Figure 3a**). To test whether reduced PTEN expression has functional relevance *in vivo*, we performed immunofluorescence experiments to demonstrate accumulation of AKT phosphorylated at serine473 (AKT-S473), a well-characterized substrate of PTEN phosphatase activity.^34^ Remarkably, motor neurons transduced with scAAV9-siPTEN displayed reduced PTEN expression and increased phospho-AKT-S473 fluorescence compared to those of scAAV9-scambled-siPTEN controls (**Figure 3b**).

### scAAV9-siPTEN is associated with increased motor neuron survival

We hypothesized that the effect on survival and phenotype seen after scAAV9-siPTEN delivery was due to increased survival of motor neurons. To assess motor neuron survival, lumbar spinal cord sections were stained for the motor neuron–specific marker calcitonin gene-related peptide (CGRP) (**Figure 3a**). Counting the number of CGRP-positive cells in lumbar spinal cord sections revealed a significant increase in motor neurons in spinal cord sections of scAAV9-siPTEN–injected mice compared to scAAV9-scrambled-siPTEN–injected controls (**Figure 3c**). Analysis of GFP-positive motor neurons revealed that the increase in total motor neuron number (**Figure 3c**) was due to an increase in GFP-positive motor neurons (**Figure 3d**) as the number of GFP negative motor neurons was not significantly different between siPTEN and scrambled-siPTEN groups (**Figure 3e**).

### scAAV9-mediated siRNA delivery reduces PTEN expression in skeletal and heart muscles

scAAV9 has been shown to transduce many organs following intravenous injection, including skeletal and heart muscle.^12,13,14^ This may be of direct therapeutic relevance for SMA as cardiac defects have also been reported in SMA patients and mouse models.^4,12,13,14^ We therefore used immunofluorescence to investigate the level of AAV9-mediated transduction in cardiac tissue; actin was labeled with rhodamine phalloidin (red), nuclei were labeled with Hoechst (blue), and GFP expression identified scAAV9-siPTEN-transduced cells. Immunofluorescence analysis revealed widespread transduction of cardiac cells.

**Figure 4:**
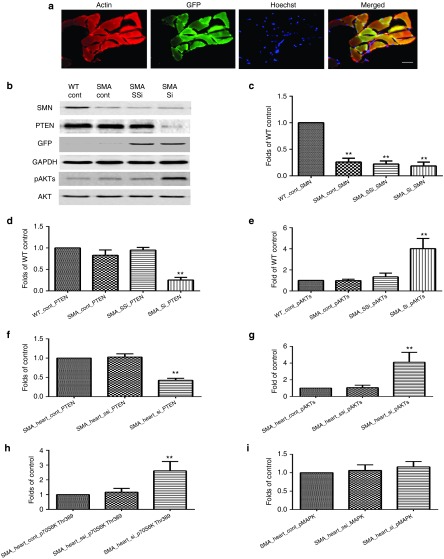
**PTEN depletion in the heart and skeletal muscle following systemic delivery of scAAV9-siPTEN in P1 SMNΔ7 transgenic mice.** (**a**) Immunofluorescence showing GFP expression in scAAV9-siPTEN-transduced heart cells (GFP, green), Hoechst labeled nuclei (blue), and actin (rhodamine phalloidin, red). Bar = 20 µm. (**b**–**e**) Western blotting in skeletal muscles. (**f**–**i**) Western blotting analysis in heart muscles.

To explore the impact of PTEN depletion on downstream pathways, we performed western blotting experiments in skeletal muscle to determine levels of AKT phosphorylated at serine473 (AKT-S473), a well-characterized substrate of PTEN phosphatase activity.^31^ Indeed, skeletal muscles from mice injected with scAAV9-siPTEN displayed reduced PTEN expression and increased phospho-AKT-S473 compared to those of scAAV9-scambled-siPTEN controls (**Figure 4b**,**d**,**e**). The level of PTEN and phosphorylated AKT was similar in wild type and untreated SMA mice (**Figure 4b**,**d**,**e**). Interestingly, SMN protein levels were unchanged in scAAV9-siPTEN mice compared to controls. Similarly, reduced PTEN expression and increased AKT and S6K phosphorylation were also observed in heart muscles of mice injected with scAAV9-siPTEN (**Figure 4f**,**g**,**h**); however, no significant change was seen in phosphorylation of MAPK (**Figure 4i**).

## Discussion

We have previously shown that depleting PTEN protein levels improves survival and growth cone size in cultured primary *Smn*^-/-^ motor neurons.^16^ Furthermore, intramuscular administration of AAV6-siPTEN to an SMA mouse model resulted in the successful targeting of motor neurons.^16^ Here, we demonstrate that systemic targeting of PTEN using viral vectors reduces disease severity and modestly improves survival in a mouse model of severe SMA.

To investigate the potential effectiveness of PTEN silencing as a therapeutic strategy for SMA, we first administered AAV6-siPTEN by intramuscular injection to the LAL muscle of SMNΔ7 mice in order to investigate its impact on NMJ pathology. As expected, AAV6-scrambled-siPTEN–treated animals displayed a reduced number of fully innervated NMJs in the caudal band of the LAL.^11^ In stark contrast, muscles from animals injected with AAV6-siPTEN had significantly higher numbers of intact NMJs. Thus, a single injection of AAV6-siPTEN was sufficient to reduce PTEN levels in motor neurons^16^ and was also able to have a direct impact on one of the earliest pathological events occurring in the neuromuscular system during the disease course of SMA. These findings therefore confirm the presence of a postnatal therapeutic time-window in SMA mice and show that during this window NMJ pathology can be ameliorated.^35^

These findings prompted us to investigate whether systemic depletion of PTEN could have a more substantial impact on the overall disease severity and lifespan of SMNΔ7 mice. AAV9 has previously been successfully used to deliver gene therapy for SMA,^22,28,30^ and we therefore generated an AAV9 vector expressing siPTEN to systemically silence PTEN. We found that a single injection of scAAV9-siPTEN significantly increased lifespan in SMA mice, with a threefold increase in mean survival compared to scAAV9-scrambled-siPTEN controls. Although the scAAV9-siPTEN–treated mice were smaller than their carrier littermates, their bodyweight increased steadily and became significantly greater than that of scAAV9-scrambled-siPTEN mice at day 11. Moreover, scAAV9-siPTEN–treated mice showed a gradual improvement in their ability to self-right over the first 2 weeks of their life, and by 15 days, 100% of treated animals were able to complete the test. The ability to self-right was then maintained throughout life. In comparison, scAAV9-ssiPTEN controls acted much like SMNΔ7 mice, with a maximum of 20% of animals completing the test.

A positive effect of PTEN depletion on neuronal survival has previously been demonstrated following transplantation of PTEN-deficient dopamine neurons into a Parkinson's mouse model,^36^ where PTEN-deficient neurons were less susceptible to cell death than controls. These findings correlate well with the data presented here showing that systemic depletion of PTEN can improve survival of spinal cord motor neurons in a mouse model of SMA. Depletion of PTEN has also been shown to enhance regeneration of corticospinal axons following spinal cord injury,^37^ demonstrating that PTEN depletion can also promote axon growth *in vivo*. Furthermore, deletion of PTEN combined with deletion of SOCS3 promotes axon regeneration in adult retinal ganglion cells following crush injury.^20,38^ Our previous studies also revealed that activation of the mTOR pathway was sufficient to trigger protein translational regulation leading to robust axonal growth as assessed by an increase in β-actin protein levels and that modulation of the PTEN/mTOR pathway also restores the specific pathological effects in motor neurons from a mouse model of SMA.^16^

There is a risk, from a therapeutic perspective, that PTEN knockdown could result in broad alteration of normal cellular activities, with the potential for tumorigenesis and/or disruption of normal neuronal function. However, the use of vectors targeted specifically to motor neurons, by using cell-specific promoters or by using a Tet on/off inducible system to finely tune PTEN knockdown, is one way to potentially overcome these issues without increased risk of tumorigenesis. Furthermore, the siRNA used in this study has been previously shown to achieve around 60% reduction in PTEN expression in motor neurons *in vitro*. Since this is not a complete depletion of PTEN, it may not be sufficient to generate unwanted off-target effects such as tumorigenesis. Thus, we speculate that the PTEN approach could be an alternative/complementary therapeutic strategy for ameliorating motor neuron pathology in SMA through a non–SMN-dependent mechanism.

Our data show that systemic depletion of PTEN can modestly improve survival in a severe SMA mouse model, suggesting a potential approach for developing therapies against SMA. Furthermore, the improvement in motor neuron survival suggests possible applications for the treatment of other neurodegenerative diseases.

## Materials and Methods

***Viral vectors.*** A 19 nucleotide sequence targeting mouse PTEN^27^ was subcloned in the scAAV9 genome vector. siPTEN sense oligonucleotide 5′-CGCGTCCCCGCCAAA TTTAACTGCAGAGTTCAAGAGACTCTGCAGTTAAATTTGGCTTTTTGGAAAT and siPTEN antisense oligonucleotide 5′-CGATTTCCAAAAAGCCAAATTTAACTGCAGAGTCTCTTGAACTCTGCAGTTAAATTTGGCGGGGA were annealed and cloned into the *Mlu*I/*Cla*I-digested vector. In addition, a scrambled (ssiPTEN) sense nucleotide 5′-CGCGTCCCCCGCAATATTCAATCGAGGATTCAAGAGATCCTCGATTGAATATTGCGTTTTTGGAAAT and ssiPTEN antisense nucleotide 5′-CGATTTCCAAAAACGCAATATTCAATCG AGGATCTCTTGAATCCTCGATTGAATATTGCGGGGGA were used to generate the control scAAV9-ssiPTEN vector. High-titer scAAV9 vectors were then prepared using a three-plasmid transient cotransfection system involving a plasmid encoding the Rep2Cap9 sequence (pAAV2/9), a helper plasmid (pHelper, Stratagene, La Jolla, CA), and the vector genomes (scAAV-siPTEN or scAAV-scrambled-siPTEN).^11^

***Animals and treatment.*** For all *in vivo* studies, FVB.Cg-Tg(SMN2*delta7)4299Ahmb Tg(SMN2)89Ahmb *Smn1*^*tm1Msd*^/J SMNΔ7 mice (The Jackson Laboratory stock 005025) were used along with carrier littermates for controls. All mice were maintained in a controlled facility in a 12-hour dark-light photocycle with free access to food and water. All *in vivo* experimental work was performed in accordance with the UK Home Office Animals (Scientific Procedures) Act 1986. Carrier animals were used for breeding, and the offspring were genotyped immediately after birth by PCR amplification of the transgenes. Animals were injected into the facial vein the day after birth (postnatal day 1) under isoflurane anesthesia with 10 µl (10^10^ vector genome) of either scAAV9-siPTEN (*n* = 10) or scAAV9-scrambled-siPTEN (*n* = 10). The animals were left to recover before being rolled in sawdust from their cage and returned to the cage with their mother. No episodes of litter exclusion were observed. Littermates were split equally between treatment groups when possible. Mice were weighed and assessed for motor ability daily as well as being scored for signs of distress. The righting reflex test was used to assess motor function as characterized previously.^39^

***Immunohistochemistry.*** Mice were terminally anesthetized with pentobarbital and transcardially perfused with phosphate-buffered saline (PBS) supplemented with heparin followed by perfusion with 4% paraformaldehyde. Relevant organs were collected and postfixed in 4% paraformaldehyde for 24 hours at 4 °C. Spinal cords and brains were cryoprotected in 30% sucrose before being cryoembedded in optimum cutting temperature medium. Following preparation of 20 µm spinal cord sections using a sliding cryostat microtome, triple immunofluorescence was performed to assess the reduction of PTEN expression in transduced motor neurons. Briefly, sections were stained using a mouse antibody to PTEN (sc-7974, Santa Cruz Biotechnology, Dallas, TX) with a goat antimouse IgG Alexa Fluor 350 Conjugate (Invitrogen, A11045 Paisley, UK) secondary antibody, a rabbit antibody to CGRP with an Alexa Flour 568-conjugated goat antirabbit secondary antibody (Invitrogen, A11036) and a goat antibody to GFP with a donkey antigoat secondary antibody fluorescein isothiocyanate conjugate (Jackson 705-095-003, West Grove, PA). To assess motor neuron survival, lumbar spinal cord sections were stained with a rabbit antibody to CGRP with an Alexa 568-conjugated goat antirabbit secondary antibody (Invitrogen A11036), and the number of CGRP-positive cells was counted. The average number of motor neurons per section in 12 sections per animal, 3 mice per group were counted in a blind manner. Heart muscles were stained with rhodamine phalloidin for F-actin (red, 1:200, Life Technologies, Paisley, UK).

***Western blot analysis.*** Fresh skeletal muscles or heart were dissected, snap-frozen in liquid nitrogen, and stored at −80 °C before analysis. Protein extraction for western blotting was performed as described previously.^15,16^ Primary antibodies, anti-mouse GAPDH antibody (1:5000; Calbiochem, Hertfordshire, UK), anti-rabbit PTEN (1:1000; Cell Signaling, Danvers, MA), AKT (1:1000; Cell Signaling), pAKT ser473 (1:1000; Cell Signaling), pS6K (1:1000; Cell Signaling), pMAPK(1:1000; Cell Signaling), goat antirabbit or mouse horseradish peroxidase secondary antibodies (1:5,000; Cell Signaling) were used.

***NMJ pathology.*** A single intramuscular injection of AAV6 expressing siPTEN (10^10^ viral particle) was delivered to the LAL muscle (from the cranial muscle group located between the ears of the mouse) in anesthetized SMA mice and control littermates at P1. Following sacrifice at P10, NMJ immunohistochemistry was performed on whole-mount muscle preparations as previously described.^13^ Briefly, the LAL was dissected in 0.1 mol/l PBS before exposure to α-bungarotoxin conjugated to tetramethyl-rhodamine isothiocyanate (TRITC-a-BTX; 5 mg/ml; Molecular Probes, Paisley, UK) for 10 minutes and subsequent fixation in 0.1 mol/l PBS containing 4% paraformaldehyde (Electron Microscopy Supplies, Hatfield, PA) for 15 minutes. Muscles were blocked in 4% bovine serum albumin and 1% Triton X in 0.1 mol/l PBS for 30 minutes before incubation with primary antibodies directed against 150 kDa neurofilament proteins (1:350 dilution; Chemicon International, Billerica, MA) overnight, followed by incubation for 4 hours in a 1:40 dilution of swine antirabbit secondary antibody conjugated to the fluorescent label fluorescein isothiocyanate (Dako, Cambridgeshire, UK). Muscles were then whole-mounted in Mowoil (Calbiochem) on glass slides before imaging on a laser scanning confocal microscope (40× objective; 0.8NA; Zeiss 710). A minimum of 50 endplates, selected at random, were assessed in each muscle preparation. To assess levels of denervation, the occupancy of individual NMJs was evaluated by categorizing endplates as either fully occupied (neurofilament entirely overlies endplate), partially occupied (neurofilament partially covers endplate), or vacant (no neurofilament overlies endplate). Four mice were used for NMJ analysis in each group.

***Statistical analysis.*** Power analysis was conducted using GPower 3.0 software (Oakland, CA), and statistical analysis was performed using GraphPad Prism v5. (La Jolla, CA) Statistical significance was determined by one- or two-way analysis of variance depending on individual experiment as stated in figure legends.

**SUPPLEMENTARY MATERIAL** **Video S1.** Video was taken on day 13. The biggest mouse is a littermate control. The medium size one is siPTEN treated. The smallest one is scramble (ssiPTEN) treated control. **Video S2.** Video was taken on day 34. The bigger mouse is a littermate control. The smaller one is siPTEN treated. No scramble control was alive at day 34.

## Supplementary Material

Supplementary Videos S1 Video was taken on day 13. The biggest mouse is a littermate control. The medium size one is siPTEN treated. The smallest one is scramble (ssiPTEN) treated control.

Supplementary Videos S2 Video was taken on day 34. The bigger mouse is a littermate control. The smaller one is siPTEN treated. No scramble control was alive at day 34.
